# Supplementary material for: Efficacy of cotrimoxazole (Sulfamethoxazole-Trimethoprim) as a salvage therapy for the treatment of bone and joint infections (BJIs)
Source: PLoS One. 2019 Oct 17;14(10):e0224106. doi: 10.1371/journal.pone.0224106 (PMC6797119; doi:10.1371/journal.pone.0224106)
Supplement: Attachment — Submitted filename: PONE.docx [file pone.0224106.s001.docx]

PONE-D-19-17215
Efficacy of cotrimoxazole (Sulfamethoxazole-Trimethoprim) as a salvage therapy for the treatment of bone and joint infections (BJI)
PLOS ONE

**First, we would like to thank the editor and the reviewer for their relevant suggestions and comments that have been considered and improved the overall revised manuscript. We hope you will find this version clarified and useful for the readers.**

==============================

It is not clear how patients were selected given that many patients may have been referred and some were treated with TMP-SMX . Please clarify what proportion of patients were treated with this agent  by and how the selection was made ie a convenience sample or some other method.

The definitions need more details and especially "diminution of CRP - was a normal value required? Please be as objective as possible.

**A: there was no normal value required; we interpreted CRP value using statistical comparison between follow-up consultation and before surgery, as supported in the result section. It now clearly appears into the text. Also, we added the number of admission in the centers during this period to clarify the volume of prescription TMP-SMX represents. Patients were treated with TMP-SMX in case they were not eligible to recommended therapy in case of an osteomyelitis. The paragraph of definitions has been completed as follows:**

- **Fifty-one patients were included in the study which stands for 3.2% of all the admission for BJI during this period.**
- **“Success was defined as the absence of local or systemic sign of infection, including delayed wound healing** **notified in the medical chart, and a statistical diminution of CRP’s value between admission and the last follow-up consultation associated with absence of relapse. Cases who did not meet above-mentioned criteria were classified as “failure”.**
- **Salvage therapy was defined by the impossibility to use recommended regimen for the treatment of BJI, notably a combination therapy with fluoroquinolones and rifampin for staphylococcal infection, either due to a resistance mechanism or intolerance. In case of polymicrobial infection or potentially resistant organism (such as ESBL or cephalosporinase), TMP-SMX was prescribed in combination therapy mainly to prevent the emergence of fluoroquinolones resistant mutants or broaden antibiotic spectrum.”**

Please clarify within the manuscript the number of cases considered resistant or intolerant to the usual recommended regimen of a FQ  plus rifampin -and then reconcile why in some cases TMP-SM|X was combined with a FQ.

**A: It now clearly appears in results section of the manuscript as follows:**

**“Overall, 25 patients were resistant to fluoroquinolones and 5 were considered intolerant, notably among elderly patients due to the risk of confusion. “**

**As previously asked in Q1, TMP-SMX was combined with a FQ because of a resistance mechanism (ESBL or cephalosporinase) or to broaden antibiotic spectrum in case of polymicrobial infection as stated in the methods section.**

There were a large number of patients excluded due to " no data" which deserves more explanation ie was it missing or not available ?

**A: data were lacking because there was no medical chart for those patients hospitalized in surgery and no follow-up consultation.**

Please indicate if the Ethics Committee was contacted and there was a waiver of any need for consent.

**A: Yes it is now clarified as follows in the Ethics section:**

**“Ethical Committee was contacted and there was a waiver of any need for consent, linked to the retrospective nature of this study, since data have already been collected and thereafter data analyzed anonymously.”**

The susceptibility of the organisms listed should be provided to both the FQs and TMP-SMX.

**A: As now explained, all the microorganisms were susceptible to TMP-SMX, considering the method of the study, and as stated earlier 25 strains were resistant to FQ. Sorry for the confusion it now appears in the the exclusion criteria in the methods as follows “being infected by a microorganism non-susceptible to SXT”**

Define multidrug resistant.

**A: MRSA (n=6) or ESBL (n=1). The sentence was unclear and withdrawn as the number of MRSA and ESBL now appears clearly in the result section and multidrug-resistant organism is defined in the methods as follows: “Multidrug-resistant organisms include ESBL-producing Enterobacteriaceae or and methicillin-resistant *Staphylococcus aureus* strains”.**

Add something to the legend in Table 2 about the microbes and that they do not add to 100% given the multiple isolates.

**A: We thank you the reviewer for his finesse. This is a great suggestion and now appears in the legends.**

Please explain why some cases had dual therapy with both a FQ and TMP-SMX. What was the rationale?

**A: We believe it is now clearer in the manuscript due to the modification of the manuscript. See answers above.**

Please tell us the normal values for the CRP and how many patients achieved a normal CRP.

**A: Normal value of CRP was <5mg/L. We added a paragraph as follows when discussing about the CRP between admission and discharge:**

**“In our center, CRP value was considered normal ≤5 mg/L and concerned 21 (41.2%) patients at the last follow-up consultation.”**

As mentioned by the peer reviewer the use of intention to treat and per protocol is usually reserved for RCTs so please rephrase this or if some other terms are used explain and introduce them in the Methods.

**A: We rephrased the paragraph because it was improper and we apologize for that. We are now talking about “those who were lost to follow-up”.**

Please define what represents hepatitis, diarrhea and cutaneous rash in more explicit terms so the readers can determine what is mean by the terms.

**A: it has been rephrased in more explicit terms as follows:**

**“in mild hepatitis (cytolysis up to 3 times above the normal value), short duration watery diarrhea, maculopapular skin rash”**

There are a large number of typographical and grammatical errors which need correcting throughout the manuscript.

The references have many errors and there are case issues which are not consistent in the listing of the references and no Latin terms have italics eg names of microbes

**A: The manuscript has been entirely proofread by an American native speaker and should be now more pleasant to read. We doubled-checked the references and the order of the references which were correct. Despite the fact we used Mendeley for the references and selected PloS One style, Latin terms were not italics. We corrected this manually.**

==============================
Journal Requirements:
When submitting your revision, we need you to address these additional requirements.

Please ensure that your manuscript meets PLOS ONE's style requirements, including those for file naming. The PLOS ONE style templates can be found at

<http://www.journals.plos.org/plosone/s/file?id=wjVg/PLOSOne_formatting_sample_main_body.pdf> and <http://www.journals.plos.org/plosone/s/file?id=ba62/PLOSOne_formatting_sample_title_authors_affiliations.pdf>

1. Thank you for including your funding statement; "no"

a. Please provide an amended Funding Statement that declares *all* the funding or sources of support received during this specific study (whether external or internal to your organization) as detailed online in our guide for authors at <http://journals.plos.org/plosone/s/submit-now>.

b. Please state what role the funders took in the study.  If any authors received a salary from any of your funders, please state which authors and which funder. If the funders had no role, please state: "The funders had no role in study design, data collection and analysis, decision to publish, or preparation of the manuscript."

 Please include your amended statements within your cover letter; we will change the online submission form on your behalf.

2. Thank you for including your competing interests statement; "no"

Please complete your Competing Interests on the online submission form to state any Competing Interests. If you have no competing interests, please state "The authors have declared that no competing interests exist.", as detailed online in our guide for authors at <http://journals.plos.org/plosone/s/submit-now>

 This information should be included in your cover letter; we will change the online submission form on your behalf.

**A: Done.**

3. We note that you have included the phrase “data not shown” in your manuscript. Unfortunately, this does not meet our data sharing requirements. PLOS does not permit references to inaccessible data. We require that authors provide all relevant data within the paper, Supporting Information files, or in an acceptable, public repository. Please add a citation to support this phrase or upload the data that corresponds with these findings to a stable repository (such as Figshare or Dryad) and provide and URLs, DOIs, or accession numbers that may be used to access these data. Or, if the data are not a core part of the research being presented in your study, we ask that you remove the phrase that refers to these data.

**A: Sorry for that, it has been removed to avoid any confusion in the findings.**


Reviewer #1: This is an interesting observational paper but the methods and limited data don't allow confidence in the conclusions proposed. The majority of patients received dual drug therapy which makes conclusions about the efficacy of cotrimoxazole alone for these infections questionable.

Q1/ A 10% loss to follow-up could substantially affect success rates and I am concerned about an additional 9 patients excluded due to duration of therapy less than 10 days and 5 excluded due to suppressive therapy. The reasons for therapy less than 10 days are not provided - was this a reflection of treatment failure? How it was determined in a retrospective chart review that treatment was for suppression rather than curative therapy is also not made clear either. These 15 patients could represent additional treatment failures but this can't be determined from the information available.

**A: Reviewer is correct we had a high level of lost to follow-up as discussed in the discussion section, maybe because those individuals are followed in multiple centers with multiple specialists (especially physiotherapist) in rehabilitation centers. However, we just describe the real situation in real life. We were concerned about your suggestions and we clarified the reasons why patient had 10 days of therapy which was not because of a failure, you can be reassured. It now clearly appears in the methods section of the revised manuscript.**

Success criteria are listed as "the absence of local or systemic sign of infection, including delayed wound healing, and the diminution of CRP and the absence of relapse". It isn't clear how in a retrospective chart review a determination was made about the "local or systemic signs of infection" or "delayed wound healing". What was the quality of documentation on these features in the charts reviewed? How was relapse defined? How subjective was this analysis? Were there specific definitions based on what was found in the charts for these criteria?
The small sample size combined with 10% loss to follow-up and the 15 excluded patients above combined with the retrospective nature of the review and possibly very subjective criteria for success when determined in a chart review make any conclusions suspect.
**A: We agree with that and it represents the major limit of our findings, but we believe that the complementary definitions and data we added to the manuscript now ensure the quality of the work. As asked by the editor, this paragraph has been rephrased. The delayed wound healing and CRP were notified in the medical chart during the follow-up consultations (see answer 1 to the editor).**

The conclusion states "SXT appears to be effective for treatment of BJI as a salvage therapy" and in description salvage therapy was referred to as "the impossibility to use recommended regimen for the treatment of BJI, notably a combination therapy with fluoroquinolones and rifampin, either due to a resistance mechanism or an intolerance."
**A: We did not understand reviewer’s question. Indeed, SXT seems to be useful in case the dual therapy FQ and RFP is compromised. We propose to use SXT as the second agent, independently of the first molecule chosen.**

The paper fails to provide adequate data to explain why cotrimoxazole might have been chosen in these patients and specifically states that "allergy was not the main reason for prescribing SXT since only a few patients presented allergy to FQ and none to rifampicin" and further suggests that the reason cotrimoxazole was chosen was not necessarily stated in the medical records.

**A:** **This question has been addressed in accordance to editor suggestion and his demand for clarifications. You will find a new paragraph with new explanations in response to your queries.**

Normally salvage therapy implies a failed trial of usual therapy before a switch to the "salvage" therapy. This is not clarified in the paper. Neither the definition of salvage therapy nor the description of why cotrimoxazole was chosen are appropriate to reach the conclusion stated.

**A: salvage therapy is defined line 110 of the revised manuscript. Complementary information on why cotrimoxazole has been chosen and appropriate are now indicated along the whole manuscript (methods and results sections).**

Use of terms "intention to treat" and "per protocol" in description of the analysis is inappropriate as it implies a prospective randomized experimental design rather than a retrospective review. There was no "protocol" when the patients were started on therapy.

**A: You are correct. We apologize for the confusion. We totally rephrased this paragraph and now as stated to the editor we talk about “individuals who were lost to follow-up”.**
