# Supplementary material for: Efficacy of cotrimoxazole (Sulfamethoxazole-Trimethoprim) as a salvage therapy for the treatment of bone and joint infections (BJIs)
Source: PLoS One. 2019 Oct 17;14(10):e0224106. doi: 10.1371/journal.pone.0224106 (PMC6797119; doi:10.1371/journal.pone.0224106)
Supplement: Attachment — Submitted filename: rebuttal.docx [file pone.0224106.s002.docx]

PONE-D-19-17215R1
Efficacy of cotrimoxazole (Sulfamethoxazole-Trimethoprim) as a salvage therapy for the treatment of bone and joint infections (BJIs)
PLOS ONE

Dear Dr. Davido,

Thank you for submitting your revised manuscript to PLOS ONE which has been substantially improved . However there remain a number of issues, mainly grammatical and syntax that need correcting. Therefore, we invite you to submit a revised version of the manuscript that addresses the points raised below.

==============================

**First, we would like to thank the editor (John Conly) for his finesse and suggestions of clarification. All the comments have been taken into consideration. We believe this version is now ready for publication.**

**Abstract**

- Should be “ a broad spectrum of activity…”

- The sentences “ We noted 76% of  orthopedic device infection (ODI). Infections were mostly polymicrobial (41%), including 47 % of gram-negative bacilli (GNB). “ and “ The drug associated with SXT was not an independent factor of success (p=0.97).”  are grammatically poor and need correcting

**We rephrased it as follows: “Gram-negative bacilli (GNB) were involved in 47% of BJIs (n=24). Moreover, they were often polymicrobial infections (41%)”** And

**“The second agent combined with SXT was not an independent factor of favorable outcome (p=0.97).”**

Prefer wording of  “ alternative oral  regimen in BJIs.” In the last sentence

**Corrected**

**Introduction**

 Better wording would be as follows in the sentences containing the following phrases

  “ ….concern in the  context of an increase ….”

“ to efficiently treat BJIs “

“  p450 ctyochromes “

“ a  major drawback of prolonged FQ administration is the”

Therefore, alternative drugs to FQs are needed. (remove the remainder of the sentence ie if we want to apply a case

“The  efficacy of SXT  in BJIs was first reported in the early  early 1970s

“ …………when other  recommended oral  agents cannot be prescribed.”

 Please use italics for Latin terms eg organism names throughout the manuscript ie *Staphylococcus* species

**Corrected**

**Methods**

 Better wording would be as follows in the sentences containing the following phrases

 “ ………..were administered empiric broad spectrum intravenous

antimicrobial therapy post-operatively combining ……”

 All adults patients should be “ all adult patients “

Please spell out the abbreviated ODI in full for first time use in the body of the manuscript

**Corrected**

Should be “……..or systemic signs of infection …” rather than sign

Correct to  “ ….wound healing recorded in the medical chart,……”

Should be “ diminution of the  CRP value…. “

Should be “ … defined by the inability to use a recommended ….”

**Corrected**

Should be “ … or potentially resistant organism (such as ESBL or

 cephalosporinase producing  organisms…..   broaden the antibiotic spectrum.

Should be “ ESBL-producing Enterobacteriaceae and/or ….”

Should be “ empiric” rather than empirical

Should be “The local Ethics Committee was contacted ………”

**Corrected**

**Results**

 Should be “ patients were included in the study which represents …. “

 Should be “…..(n=14) and 12% methicillin-resistant…”

Should be worded better as  “ Overall, 25 patients had organisms which were resistant to fluoroquinolones and 5 patients  were considered intolerant to the oral agents,  notably among elderly patients related to confusional states. “

Should be “……..surgery during  the course of therapy.”

Should be “ ……………. The procedures consisted of …….”

Should be “ ………….was performed in 26%  (n=8) of cases vs debridement….”

Should be “…. Table 3. Surgical management and antimicrobial regimen.’

Same for the subtitle – better as Antimicrobial Regimens “

**Corrected**

Please use another term other than “relay” for the use of an oral regimen

**We simply used the term oral regimen.**

Should be “In our center, the CRP value was considered normal at a value of  ≤5

 mg/L and was found in 21 (41.2%) patients at the last follow-up consultation.”

Should be “………….not significantly associated……………..”

Should be “Adverse events consisted of  a mild hepatitis (values up to 3 times

above the normal), a short duration watery diarrhea,  a cutaneous maculopapular skin rash and an isolated fever in 1 case each. No renal failure was diagnosed during follow-up.

 SXT was interrupted in 6% (n=3): 1 patient had replacement with a switch to another antimicrobial therapy (n=1) and 2 antibiotic discontinuations were noted. “

**Corrected**

**Discussion**

 Better wording would be as follows in the sentences containing the following phrases

 Should be “ This retrospective study documents numerous patients treated with a

combination therapy based on SXT for the treatment of a polymicrobial BJI”

 Should be “ ………. can appear disappointing but it rises up to 87%  in the best case scenario.”

 Should be “ This rate is gratifying  if we consider the higher proportion than usual of these particular prosthetic joint infections due to polymicrobial and Gram negative bacteria.”

 Should be “  Moreover it concerns elderly and fragile patients with frequent comorbidities who underwent previous surgeries which failed initial therapy and therefore required the expertise of a reference center.”

 Should be “ This latter study showed similar  outcomes using this regimen based on SXT versus………..”

 Should be “ In addition, median duration of treatment was 6 weeks which is generally compliant according to the French guidelines [22] and promoting the appropriate use of antibiotic duration.”

**Corrected**

 Should be “ Likewise, although costs have not been studied, some  literature has already reported a diminution of costs when…….” And there should be a reference added to support .

**References were added, sorry for that. It was a problem with the software (Mendeley).**

The phrase “………but also than the ones reported in previous cohorts of BJI treated with SXT………… “ is missing something – please correct

**We rephrased it as follows: “but also than the ones reported in previous cohorts of BJIs treated with SXT which preconized high doses [27,29–31].”**

Should be “ ………. For  antibiotic selective pressure,”

Eliminate the sentence “A particular attention to renal function and potassium level must be paid in these patients especially when using high dosing of SXT. “ It adds very little and helps to tighten the manuscript.

**Done**

**Conclusion**

 Eliminate “……….in common daily encountered practice………….”

**Withdrawn.**

**References**

 There are a few items to correct with respect to case in the references eg the last 2 and other refs have the title of the paper all in capital letters – please correct

**Corrected.**

==============================
