# Supplementary material for: Efficacy of cotrimoxazole (Sulfamethoxazole-Trimethoprim) as a salvage therapy for the treatment of bone and joint infections (BJIs)
Source: PLoS One. 2019 Oct 17;14(10):e0224106. doi: 10.1371/journal.pone.0224106 (PMC6797119; doi:10.1371/journal.pone.0224106)
Supplement: Attachment — Submitted filename: rebuttal-R2.docx [file pone.0224106.s003.docx]

PONE-D-19-17215R2
Efficacy of cotrimoxazole (Sulfamethoxazole-Trimethoprim) as a salvage therapy for the treatment of bone and joint infections (BJIs)
PLOS ONE

**First, we would like to thank the editor (John Conly) for his proofreads and suggestions of clarification. All the comments have been taken into consideration. We believe this version is now ready for publication.**

Abstract

Should be "..... a broad spectrum of activity with adequate...."

**Already written as suggested.**

"We noted 76% of orthopedic device infection (ODI)."  is a grammatically poor sentence and suggest to add the finding into the first sentence to avoid 2 short choppy sentences. Also in Table 1 you indicate that the age 60 was a median but you indicate a mean in the abstract - please correct  and also add in brackets the SD in the abstract ie " We analyzed 51 patients with a ?  mean ? median  age of 60 ± 20  (SD) years of which 76% presented with an orthopedic device infection (ODI).

**Thank you for the example. Actually median was=median so it is another way to show our results. Usually mean are commonly used in abstract, therefore we kept this presentation of our findings.**

Introduction

Should be "..... due to a broad spectrum of activity with good bone diffusion...."

**Corrected, thank you.**

Italics for *Staphylococcus spp* was forgotten to be added

**It’s now in italics, sorry for that mistake.**

The genus for *Clostridium* has been officially changed to *Clostridioides* so please change throughout the text where used but not the references

**Corrected.**

Should be " leading to the suggestion of SXT.... "

**Corrected.**

Methods

Should be ".....SXT use, route of administration..... " rather than currently shown as "SXT use: route of administration

**“:” has been changed to “,”**

The sentence ending with "associated drugs , "  ends with a  comma rather than a period.

**It was changed to a period as requested.**

Results

Note the discrepancy of use of a median age as in Table 1 as opposed to mean in the abstract - please correct

**Median is used in the Table 1 to illustrate IQR as we are dealing with a small sample size study. As clarified above, Median was equal to mean in our work.**

 Discussion

Why do you say " .......... previous surgeries which failed failure  initial therapy ....." - please clarify or correct

**Sorry for the duplicate and repeated word namely “failed failure”. Sentence was also clarified because it was referring to table 1: “previous surgeries which failed initial therapy (as illustrated in Table 1) and therefore required the expertise of a reference center.”**

Please correct the sentence " ...Harbarth et al described 85.7% of success ...." do you mean " a 85.7 % success rate...."

**Yes, sorry for that misunderstood. It is now corrected.**

Prefer to say " "A combination of therapy of SXT with a  FQ in these patients was associated with a favorable outcome in 82.3% ." than as currently written.

**Thank you for the correction, which has been applied.**

".... preserve FQs from acquired resistance." should be "...... FQs acquiring resistance"

**Thank you for the correction, which has been applied.**

Something is incomplete or erroneous in the sentence ".... but also than the ones reported in previous  cohorts of BJIs treated with SXT which preconized...." - please correct

**Sorry the word lower was missing in our sentence. We also shortened the sentence because the end was obvious, as follows: “the doses of SXT we used were frequently lower than recommended by the French (3200/640mg) and US guidelines (8 mg/kg of trimethoprim), but also lower than the ones reported in previous cohorts of BJIs treated with SXT”**

Should be plural ie "side effects "

**Corrected.**

Should be " SXT is responsible for various ..." rather than "... of various "

**Thank you for the correction, which has been applied.**

Should be " .... reinforces the usefulness of SXT"

**Sorry for the typo, it has been corrected.**

Please rephrase " deadend situations " as not a scientific term

**It has been changed to “deadlock” which can be used in scientific term, especially for antibiotics. (ie :** <https://www.ncbi.nlm.nih.gov/pubmed/24698433>)

Should be plural ie    ".... physicians should .... "

**Corrected.**

Conclusion

Rather than ".... taking into consideration such molecule" why not just say " taking into consideration the use of SXT" since that is what your article suggests

**Thank you for the suggestion which clarified the conclusion and has been applied.**
